# The impact of burning mouth syndrome on health-related quality of life

**DOI:** 10.1186/1477-7525-9-57

**Published:** 2011-07-29

**Authors:** Fabrício TA Souza, Tálita PM Santos, Vanessa F Bernardes, Antônio L Teixeira, Arthur M Kümmer, Tarcília A Silva, Mauro HNG Abreu

**Affiliations:** 1 Department of Oral Surgery and Pathology, (Av. Antônio Carlos, 6627), School of Dentistry, Universidade Federal de Minas Gerais, Belo Horizonte, (Postal Code - 31270.901), Brazil; 2 Department of Internal Medicine, (Avenida Alfredo Balena, 190), Faculty of Medicine, Universidade Federal de Minas Gerais, Belo Horizonte, (Postal Code 30130.10), Brazil; 3 Department of Mental Health, (Avenida Alfredo Balena, 190), Faculty of Medicine, Universidade Federal de Minas Gerais, Belo Horizonte, (Postal Code 30130.10), Brazil; 4 Department of Community and Preventive Dentistry, Av. Antônio Carlos, 6627, School of Dentistry, Universidade Federal de Minas Gerais, Belo Horizonte, (Postal Code - 31270.901), Brazil

**Keywords:** Burning Mouth Syndrome, Quality of life, epidemiology

## Abstract

**Background:**

Burning mouth syndrome is a chronic disorder that is characterized by a burning sensation and a normal clinical appearance of the oral mucosa. This condition often affects the health-related quality of life in patients. As such, the aim of this study was to compare the health-related quality of life of patients with BMS and healthy controls, using the validated Portuguese versions of the SF-36 and OHIP-49 questionnaires.

**Methods:**

A calculated sample of Brazilian patients with BMS (n = 26) was compared with a control group (n = 27), paired for gender and age. Sociodemographic information and clinical characteristics were obtained, and interviews were conducted using the SF-36 and OHIP-49. To evaluate the normality of the variables, we used the Kolmogorov-Smirnov test. The chi-square test, Fisher exact test and Mann-Whitney U-Test were used to compare sociodemographic and clinical characteristics of individuals with BMS and controls Mann-Whitney U-test were carried out to compare SF-36 and OHIP-49 between BMS patients and controls. The significance level was set at 0.05. To compare the dimensions of the SF-36 and OHIP-49 between BMS patients and controls, we considered Bonferroni correction. So for comparison of the dimensions, the significance level was set at 0.00625 for SF-36 and at 0.00714 for OHIP-49.

**Results:**

The clinical and demographic data were similar in both groups (P > 0.05). SF-36 scores were significantly lower in all domains for patients with BMS (P < 0.00625). OHIP-49 scores were higher for individuals with BMS (P < 0.00714).

**Conclusions:**

BMS has a negative impact on the health-related quality of life of individuals, as can be shown by instruments such as the SF-36 and OHIP-49. So, the evaluation of quality of life might be useful for more information about the nature and severity of BMS, to evaluate the effects of treatment protocols, in order to improve their outcomes by means a humanized clinical practice.

## Introduction

Burning mouth syndrome (BMS) is a chronic disorder that has evolved as a distinct clinical entity [1,2]. BMS is more common in women in the middle to elderly age range [2,3]. The prevalence is estimated to be 0.7-4.6% of the general population [2]. BMS involves burning sensations in the oral mucosa without evident clinical pathology or laboratory findings [1-4]. The burning has been reported to be of moderate or severe intensity and may vary throughout the day [2,3]. Multiple factors have been associated with these changes, and a variety of symptoms could be simultaneously present, such as xerostomia, dysgeusia, and psychological dysfunction. Multiple sites in the oral cavity may be affected, with the tongue being the most commonly affected site [2,4]. Because of the lack of consensus about the etiology of BMS, establishing a treatment protocol for patients has been extremely difficult [3].

It has been shown that BMS exerts a negative impact on the quality of life of affected individuals [5-9]. According López-Jornet et al. [5], patients with BMS have poorer scores on all scales that measure quality of life. It is necessary to identify, explore, discriminate and measure the oral quality of life for individuals with BMS to devise specific strategies to improve the quality of life of these patients [5]. Assessing the quality of life of patients during the treatment of BMS may improve patient-reported outcome measures, including quality of life [6].

As such, the aim of this study was to compare the health-related quality of life of patients with BMS and healthy controls, using the validated Portuguese versions of the SF-36 and OHIP-49 questionnaires.

## Methods

### Participants

The present investigation was a cross-sectional controlled study that evaluated patients being seen in the Oral Pathology clinic of Universidade Federal Minas Gerais for the treatment of BMS symptoms. The Human Research Ethics Committee of Universidade Federal de Minas Gerais approved this study. All participants provided signed informed consent forms.

The sample size calculation was performed with α = 0.05, power (1-β) = 0.95 and mean and standard deviation values for the OHIP-49 from another study [5]. This formula resulted in 21 patients for each group. However, considering that Universidade Federal de Minas Gerais is a reference center for the treatment of BMS, we evaluated 26 cases and 27 control patients. We evaluated a consecutive sample of patients referred to the Oral Pathology Clinic, School of Dentistry, Universidade Federal de Minas Gerais and to the Oral Pathology Clinical of the Odilon Behrens Hospital, a reference public Hospital in Belo Horizonte, for the treatment of changes associated with BMS, between August 2009 and December 2010.

The inclusion criteria for the diagnosis of BMS were in accordance with those in the International Classification of Headaches, which describes the following criteria for the diagnosis, such as pain, itching, or burning in the mouth present daily and persisting for most of the day, with apparently normal oral mucosa and absence of local and systemic diseases [7].

All patients underwent diagnostic blood tests (e.g., complete blood cell count, as well as levels of glucose, iron, transferrin, vitamin B12, folic acid, antinuclear antibodies (ANA), Anti-SSA/RO and Anti-SSB/LA) in order to exclude exclude other disorders that cause burning in the mouth as Sjogren's syndrome, diabetes and anemia [1,8,9]. Two independent examiners (two experts oral pathologists) performed the clinical oral examinations to confirm the absence of oral lesions.

The control group consisted of healthy patients seen in the Dental Clinic of Universidade Federal de Minas Gerais for periodic reviews of their dental condition. They were not receiving any treatment at the time of study, and the patients in the group had no history of chronic pain syndrome or concomitant locoregional disease that caused oro-facial pain. Participants in the control group were selected for similar age and gender, on the basis of the age and gender characteristics of the subjects with BMS. All participants in the study were from the Belo Horizonte metropolitan area in Minas Gerais.

We recorded the following sociodemographic information and clinical characteristics: age, gender, work, presence of systemic diseases, use of medications, denture wear, tobacco and alcohol use and duration of symptoms.

The intensity of symptoms in patients with BMS was measured using a Visual Analogue Scale (VAS). VAS consists of a 10-cm line with two closed ends. One end indicates 'without burning' while the other end indicates 'unbearable burning sensation,' representing the opposite extreme. Patients were asked to score a single point according to the best matched burning intensity [10].

Oral interviews with individuals with BMS and with control subjectswere carried out between August 2009 and December 2010.

### Questionnaires of quality of life

To assess quality of life, we appliedtwo questionnaires: one that assesses general health (SF-36) and another that evaluates the impact of oral health on quality of life (OHIP-49).

The SF-36 is a questionnaire with 36 items across eight components: physical functioning, physical role limitations, physical pain, general medical health, vitality, social functioning, emotional role limitations and mental health. Higher scores indicate better health. This instrument assesses the impact of general health on quality of life [11]. The Brazilian standard version of the SF-36 was validated in our population [12].

The OHIP-49 consists of 49 questions organized into seven dimensions: functional limitation, physical pain, psychological discomfort, physical disability, psychological disability, social disability, and handicap. The five response categories are assigned values of 0-4 and indicate never (0), hardly ever (1), sometimes (2), fairly often (3), and very often (4) [13]. The Brazilian standard version of the OHIP-49 was validated in our population.

### Statistical analysis

We assembled a database using Statistical Package for Social Sciences (SPSS) version 18.0. The descriptive statistical analysis involved calculations of proportions, measures of central tendency and variability for the sociodemographic and clinical aspects as well as quality of life variables. To evaluate the normality of the variables, we used the Kolmogorov- Smirnov test. The chi-square test, Fisher exact test and Mann-Whitney U-Test were used to compare sociodemographic and clinical characteristics of individuals with BMS and controls Mann-Whitney U-test were carried out to compare SF-36 and OHIP-49 between BMS patients and controls. The significance level was set at 0.05. To compare the dimensions of the SF-36 and OHIP-49 between BMS patients and controls without increasing in probability of type 1 error, we considered Bonferroni correction. So for comparison of the dimensions, the significance level was set at 0.00625 for SF-36 (0.05 divided by 8 dimensions) and at 0.00714 for OHIP-49 (0.05 divided by 7 dimensions).

## Results

The sample consisted of 26 individuals with BMS, 24 women (93.3%) and 2 men (7.7%), with a mean age of 63.62 ± 10.96 years and a median age of 64.0 years. The control group consisted of 25 women (92.6%) and 2 men (7.4%), with a mean age of 64.59 ± 11.56 years and a median age of 67.0 years. The age and gender distributions were similar in both groups (*P  *> 0.05). The demographic characteristics for the 26 subjects with BMS and the 27 control subjects are presented in Table 1.

**Table 1:** Comparisons of sociodemographic and clinical characteristics of individuals with BMS and controls, Brazil, 2009-2010

	BMS	Control	P-value
Age (years)	64.0 (Median)	67.0 (Median)	0.75*
Women	24 (93.3%)	25 (92.5%)	1.0***
Work outside the home	9 (34.6%)	8 (29.6%)	0.69**
Systemic Diseases	25 (96.2%)	23 (85.2%)	0.35***
Medication use	24 (92.3%)	22 (81.5%)	0.42***
Antidepressive medication	10 (38.5%)	5 (18.5%)	0.11***
Antihypertensive medication	13 (50.0%)	18 (66.7%)	0.22***
Smoking	3 (11.5%)	2 (7.4%)	0.67***
Alcohol	2 (7.7%)	0 (0.0%)	0.23***
Dentures Wearers	16 (61.5%)	16 (59.3%)	0.86**

* Mann-Whitney U-Test ** Chi-square Test *** Fisher Exact Test.

The mean duration of BMS symptoms was 37.85 ± 43.13 (1-180) months. Among the subjects with BMS, symptoms were measured using the VAS, yielding a mean score of 8.81 ± 1.69.

SF-36 scores showed that the subjects with BMS, when compared with the control group, had significantly lower median scores across all of the domains (P < 0.00625) (Table 2).

**Table 2:** SF-36 scores for 26 individuals with BMS and 27 healthy controls, Brazil, 2009-2010

SF-36	BMS Median (Min-Max)	Control Median (Min-Max)	P-value*
Physical Function	57.5 (5.0-100.0)	80.00 (40.0-100.0)	< 0.001
Physical Roles	50.0 (0.0-100.0)	100.00 (0.0-100.0)	0.006
Physical Pain	50.5 (0.0-100.0)	72.00 (10.0-100.0)	0.003
General Health	63.5 (5.0-100.0)	87.00 (12.0-100.0)	< 0.001
Vitality	27.5 (0.0-95.0)	75.00 (5.0-100.0)	< 0.001
Social Functioning	50.0 (0.0-100.0)	87.50 (50.0-100.0)	< 0.001
Emotional Roles	66.6 (0.0-100.0)	100.00 (0.0-100.0)	< 0.001
Mental Health	36.0 (0.0-100.0)	76.00 (32.0-100.0)	< 0.001
SF-36 (all items)	46.9 (11.1-94.4)	83.19 (23.4-100.0)	< 0.001

*Mann-Whitney U-Test.

Regarding the OHIP-49, we found higher scores for individuals with BMS than for the control group. Furthermore, significant differences were found for all domains of the questionnaire (P < 0.00714) (Table 3).

**Table 3:** OHIP-49 scores for 26 individuals with BMS and 27 healthy controls, Brazil, 2009-2010

OHIP-49	BMS Median (Min-Max)	Control Median (Min-Max)	P-value*
Functional Limitation	18.58 (1.49-32.39)	7.97 (0.00-27.31)	< 0.001
Physical Pain	20.91 (7.85-37.26)	3.87 (0.00-20.01)	< 0.001
Psychological Discomfort	36.32 (7.60-37.26)	3.90 (0.00-35.98)	< 0.001
Physical Disability	16.00 (0.00-27.46)	0.00 (0.00-23.42)	< 0.001
Psychological Disability	29.07 (0.00-40.00)	2.78 (0.00-31.08)	< 0.001
Social Disability	22.13 (0.00-40.00)	0.00 (0.00-14.03)	< 0.001
Handicap	18.58 (0.00-34.31)	0.00 (0.00-17.15)	< 0.001
OHIP-49 (all items)	22.89 (4.25-33.24)	3.06 (0.00-22.02)	< 0.001

*Mann-Whitney U-Test.

## Discussion

Health-related quality of life has been a widely used instrument for assessing the physical and psychosocial impact of chronic diseases, and this measure has led to a better understanding of patients with these diseases and their conditions for adaptation [11,14]. Patients with BMS have been reported to have a diminished quality of life [5,6,15-17]. Corroborating these findings, the present study showed that BMS had a negative impact on the health-related quality of life of individuals across all domains by using instruments such as the SF-36 and OHIP-49.

BMS is a disorder with symptoms that include persistent burning sensations, xerostomia and taste disturbances [1,3], in addition to several associated systemic changes, such as psychological disorders, gastrointestinal maladies and urogenital problems [18,19]. These disorders can contribute to a diminished quality of life for these patients [5,6]. In the present study, we did not evaluate specific systemic alterations; however, no differences were observed between the clinical data of patients with BMS and the control subjects.

The SF-36 is a generic questionnaire with concepts that are not specific to a certain age, disease or treatment group; thus, the questionnaire allows for comparisons between different diseases and different treatments [11,12]. This questionnaire also includes perceptions by individuals regarding their own health status across most representative aspects of health [11]. The instrument, when applied to individuals with BMS, proved that the condition had a negative impact on the quality of life of these patients compared with healthy controls and compared with individuals with other diseases of the oral mucosa [5,15]. In line with these findings, we observed that individuals with BMS have a negative impact on all domains of the SF-36.

The OHIP-49 was developed with the aim of providing a comprehensive measure of dysfunction, discomfort and disability reported by individuals that are attributed to oral conditions [13]. In all studies on the quality of life of individuals with BMS, the OHIP-49 was used in its original form or the short form, which also showed a negative impact on the health- related quality of life of such individuals [5-9]. Corroborating these findings, the present study found that all domains negatively affected the quality of life of patients with BMS.

Burning mouth syndrome has been characterized by changes associated with multiple symptoms and persistent burning sensations [2-4,8]. Because its etiopathogenesis is still unclear regarding the probable neuropathic origins, no consensus has been established regarding effective treatment [2,3]. Fewer than 3% of patients experience a complete regression of symptoms over a period of five years [20]. Patients with BMS in our study had, in addition to their symptomatology, over three years of evolution and high scores on the VAS. These factors may have contributed to the finding that patients with BMS in our study had high scores within the OHIP-49 domains.

The scores in the Psychological Disability domain reaffirms the mutual relationship between psychiatric disorders and BMS, as described previously in other studies [2-4,19] and as also reported in the Mental Health domain of the SF-36. This association should be further investigated because it may have a negative impact on the quality of life of patients with BMS and because it intervenes directly in the biopsychosocial environment of affected individuals.

The majority (84.6%) of patients had severe burning sensations [21] and have BMS for years, what could explain the relationship between BMS and health-related quality of life.

This study presents advantages and limitations associated with a cross-sectional controlled study. Considering that we measured BMS and quality of life at the same time, our associations could not be considered as causal [22]. Besides, we have not evaluated dental caries. This disease could have affected quality of life, despite the two group of patients have had similar high proportion of dentures wearers. Among the strengths of this study was the presence of a calculated sample in a reference center for the treatment of disease.

## Conclusions

BMS has a negative impact on the health-related quality of life of individuals, as can be shown by instruments such as the SF-36 and OHIP-49.

So, the evaluation of quality of life might be useful for more information about the nature and severity of BMS, to evaluate the effects of treatment protocols, in order to improve their outcomes by means a humanized clinical practice.

## Competing interests

The authors declare that they have no competing interests.

## Authors' contributions

FTAS carried out the data collection, participated in the conception and design of this study, analyzed and interpreted the data. TPMS and VFB carried out the data collection and analysis of data. ALT and AMK participated in the conception and design of this study, helped on analysis and interpretation of the data. TAS and MHNGA conceived of the study, participated in its design and coordination, analyzed and interpreted the data. All authors helped to draft the manuscript, read and approved the final manuscript.
